# Using sentiment analysis to evaluate the impact of the COVID-19 outbreak on Italy’s country reputation and stock market performance

**DOI:** 10.1007/s10260-023-00690-5

**Published:** 2023-04-03

**Authors:** Gianpaolo Zammarchi, Francesco Mola, Claudio Conversano

**Affiliations:** Department of Economics and Business Science, University of Cagliari, Cagliari, Italy

**Keywords:** Sentiment analysis, Twitter, COVID-19, Stock market performance, Country reputation, Machine learning, MSC 62

## Abstract

During the recent Coronavirus disease 2019 (COVID-19) outbreak, the microblogging service Twitter has been widely used to share opinions and reactions to events. Italy was one of the first European countries to be severely affected by the outbreak and to establish lockdown and stay-at-home orders, potentially leading to country reputation damage. We resort to sentiment analysis to investigate changes in opinions about Italy reported on Twitter before and after the COVID-19 outbreak. Using different lexicons-based methods, we find a breakpoint corresponding to the date of the first established case of COVID-19 in Italy that causes a relevant change in sentiment scores used as a proxy of the country’s reputation. Next, we demonstrate that sentiment scores about Italy are associated with the values of the FTSE-MIB index, the Italian Stock Exchange main index, as they serve as early detection signals of changes in the values of FTSE-MIB. Lastly, we evaluate whether different machine learning classifiers were able to determine the polarity of tweets posted before and after the outbreak with a different level of accuracy.

## Introduction

Twitter is a microblogging and social media service widely used by users to interact and publish content in response to events. Twitter is being largely used to share information and express sentiment and concerns on the recent Coronavirus disease 2019 (COVID-19) outbreak, which was first identified in December 2019 in Wuhan, Hubei, China, and resulted in a serious threat to public health worldwide (Hui et al. 2020). Italy was one of the first European countries to be severely affected by the outbreak as well as to implement extraordinary measures to limit viral transmission, such as lockdowns and stay-at-home orders (Remuzzi and Remuzzi 2020). This situation might have led to extensive concerns towards Italy, potentially leading to country reputation damage, loss of investments and tourism flows. In particular, since a country’s reputation is usually studied in terms of strategic public diplomacy, effective nation-building, and nation branding (see, for example, Yang et al. 2008), it is very likely that a dramatic event like the COVID-19 outbreak negatively affects all three dimensions of the reputation of a country. In the following, we focus specifically on the third dimension (national branding) but concentrate on the reputation of Italy perceived by Twitter’s users, the latter measured through sentiment analysis.

Sentiment analysis (Pang and Lee 2008), sometimes referred to as opinion mining or polarity classification, is aimed at analyzing and classifying text into sentiments with polarity or specific emotions using different approaches. In today’s world, communication has become more and more bidirectional, involving not only a company or other entities sending messages/information to people but also vice versa. The widespread use of social media allows billions of people from around the world to have direct interaction with companies, actors/actresses, sportsmen/sportswomen, or politicians, just to give some examples. Having a tool able to understand what a text means without the need for a human interpretation is of huge value, e.g. for a company that needs to reply to thousands of e-mails, letters, messages, or any other type of communication. Social media could be a huge source of information, but the sheer number of messages exchanged every day makes it very difficult for any company or organization to extract knowledge from them in a quick way. For example, it would be useful to know that, due to a given event, many people in a specific area of the world need something. We might add that this information could be useful in different situations, e.g. a company trying to intercept the unexpressed needs of potential customers but also a humanitarian organization willing to help people in areas struck by war or natural disasters. Although these two situations can be thought of as very distant cases, they share the importance of correctly interpreting a message. This is a very hard task and sentiment analysis tackles only a part of it, as it is focused on the polarity and strength of a text. Moreover, sentiment analysis usually has two (positive, negative) or three (positive, neutral, negative) possible outcomes and can be performed at three different levels: document level, sentence level, and aspect level. At a document level, a document can be thought of as a collection of sentences (e.g. a product review or a book chapter). Each sentence is evaluated separately and then aggregated to express an overall sentiment. At a sentence level, the evaluation is made for every single sentence, resulting in a more fine-grained analysis than at the document level. Finally, at an aspect level, the analysis can be performed for every single aspect of interest. For example, in a product review, we might be interested in price, package, delivery, and many other aspects. Every aspect is independent since we can have a positive sentiment for price and a negative sentiment for a package in the same product review.

There are two main approaches to performing sentiment analysis: methods that do not rely on a model (e.g. lexicon-based approaches) and methods that use a model. The former use a list of words (lexicon) associated with a specific sentiment polarity. The sentiment of some document, sentence, or text, in general, is given by the number and strength of positively/negatively associated words in that text (Turney 2002). It is possible to use an existing lexicon or to build a new one. The other approach can instead be thought of as building a model to accomplish a supervised or unsupervised classification task. Several models/classifiers can be used, e.g. naïve Bayes (NB), Support Vector Machine (SVM), logistic regression, decision trees, neural networks, etc. They work differently and might have different performances, but they all share the same steps: collect, label (in case of supervised approaches), and process data, split the data set into training and test set, build the model on the training set, assess model performance.

In the above-described framework, the main contributions of this study are summarized as follows:

1. Analyze the temporal evolution of the sentiment towards Italy before and during the COVID-19 outbreak through sentiment analysis of tweets. Our goal is to show that right after the media started spreading the news of the first Italian case of COVID-19 the sentiment towards Italy and the perceived country’s reputation, in particular the national brand, began to drop significantly. We show, at the end of Sect. 3.2, that whilst all negative emotions increase, the positive ones have different behavior, linked to the specific type of emotion considered.
2. Compare the changes in tweets’ sentiment with the evolution of stock exchange figures, in particular with the prices of the main Italian stock exchange index (FTSE-MIB). We follow the line of research investigating the ability of sentiment analysis to predict stock market movements (see, for example, Pagolu et al. 2016) and assume the polarity of a sentiment expressed on Twitter towards Italy can reverberate on several aspects of the life of a country, including the performance of the stock market which is known to be affected by exogenous factors.
3. Evaluate whether differences in the ability of different machine learning classifiers to assess the polarity of tweets can be detected when comparing tweets posted before and after the COVID-19 outbreak. To this aim, we use two of the most popular models (NB and SVM) to perform the task of classification of tweets into positive, neutral, or negative. This task is performed separately for tweets posted in the two periods identified based on the main breakpoint in sentiment scores. While these results have to be interpreted with caution based on the limited number of labeled tweets and variability of obtained estimates, we speculate that the change in the sentiment of the tweets observed in the two periods might also reflect differences in the classification performance. We hypothesize that tweets posted in the period following the outbreak might be slightly easier to classify based on the fact that they are less heterogeneous and more focused on topics or sentiments related to the ongoing spread of the pandemic, as well as on the negative consequences it generates for the country.

The rest of the paper is organized as follows. Section 2 introduces previous studies performing sentiment analysis on tweets in response to disease outbreaks. Section 3 describes lexicon-based sentiment analysis to identify shifts in the sentiment towards Italy after COVID-19 outbreak. In Sect. 4, shifts in sentiment polarity are compared with changes in stock exchange market performance. Performances of two machine learning classifiers in the classification of tweets are compared in Sect. 5 and the conclusions of the study are presented in Sect. 6.

## Literature review

A number of studies performed sentiment analysis on Twitter data in response to important events. This kind of analysis is considered a good way to measure public opinion since users are free to express their thoughts about any topic having a (potentially) large audience (see, for example, Paul and Dredze 2017; Tavazoee et al. 2020). Here, we focus on those related to response to disease outbreaks.

Chunara et al. (2012) analyze data from news media, Twitter, and official reports by the government during the first 100 days of the 2010 Haitian cholera outbreak. Data about trends in the volume of informal sources is significantly correlated with official case data and is available up to two weeks earlier, thus being potentially useful to provide timely estimates of outbreak dynamics.

Similarly, in 2010 Chew and Eysenbach (2010) collect about two million tweets related to the influenza outbreak and show that sentiment analysis performed on Twitter data is a valid tool to measure public perception, allowing health authorities to address real as well as perceived concerns. This is particularly important considering that misinformation might allow a disease to spread more quickly, with a significant cost to health and human lives, while correct information might contribute to adopting behavioral changes (e.g. social distancing), especially in the initial stage of an outbreak when a vaccine is not available. Signorini et al. (2011) show other ways in which tweet analysis is useful during the outbreak. Specifically, the extraction of information from a live stream of tweets allows early detection of hot spots. Importantly, Szomszor et al. (2010) collect about three million tweets and use them to detect trends of infection spreading one week earlier compared to the official reports, showing how Twitter data analysis is useful as an early warning detection system. Smith et al. (2016) analyze tweets posted during the flu season in the US in 2012/2013. They use machine learning models to separate tweets about flu awareness from tweets about the infection, demonstrating that these two types of tweets show different trends. For instance, they document that levels of awareness drop after the peak, even when infection levels are still high. Using a different approach, Broniatowski et al. (2013) analyze Twitter data posted during the same flu season to build a model able to distinguish between tweets reporting an infection from genetic tweets mentioning flu. Using this tool, they are able to predict changes in influenza prevalence with an accuracy of 85%.

Starting in 2014, several studies used Twitter data to analyze reactions to the Ebola outbreak in Africa. A number of these articles focus on the perception of the disease among people living in Western countries (e.g. US). Fung et al. (2014) show that despite the disease, the outbreak was far away from the US (only a few cases hit the US, while the large majority of cases were in Guinea, Sierra Leone and Liberia), people were very concerned about their own safety. This is proved by the high levels of anxiety, anger, and other negative emotions that were significantly higher than those observed during the influenza outbreak.

Other papers focus on what was happening in Africa. Oyeyemi et al. (2014) collect tweets using keywords such as “Ebola" and “prevention" or “cure" and find that many of them are carrying misleading information. They split their data into correct information, medical misinformation, and a generic category for tweets not in these two categories (other). Since some of these remedies are medically questionable (e.g. drinking salted water), and the potential audience is very large, the Nigerian government itself decided to respond to this misinformation using Twitter.

Finally, a growing number of studies used social media analysis to investigate different aspects of the COVID-19 pandemic. A large part of these studies is focused on the identification of the main topics among COVID-19-related tweets. Using Latent Dirichlet Allocation (LDA) analysis, Abd-Alrazaq et al. identify as main themes those related to the origin of the virus, its impact on the economy as well as actions to mitigate the risk of infection (Abd-Alrazaq et al. 2020). Among studies not restricted to the English language, Garcia and Berton compare the main topics among COVID-19-related tweets written in English and Portuguese, showing similar patterns and a prevalence of negative emotions related to the proliferation of care options, case reports and statistics (Garcia and Berton 2021). Other studies focus on relevant problems such as misinformation, fake news, and conspiracy theories in relation to COVID-19 vaccines. Daradkeh analyzed 40,359 tweets related to COVID-19 vaccination, collected between January 2021 and March 2021, showing that, across different misinformation topics, the average number of replies, retweets, and likes of tweets with negative sentiment is higher compared to those with positive sentiment (Daradkeh 2022). Ahmed et al. perform an analysis of tweets for a 7-day period (March 27, 2020, to April 4, 2020) in which the $\#$5GCoronavirus hashtag was trending in the United Kingdom (Ahmed et al. 2020). The authors report that the majority of tweets are actually posted by non-conspiracy theory supporters trying to confute the fake news, suggesting that only a minority of users using this hashtag believed the conspiracy (Ahmed et al. 2020). Other studies focus on the application of the machine or deep learning models for the classification of tweets, aiming to evaluate the public perception as regards opinions and emotions related to different aspects of the pandemic such as social distancing (Shofiya and Abidi 2021), fake news (Chintalapudi et al. 2021) or vaccination (Aygun et al. 2021). A recent study explores the relationship between emotions contained in tweets and their popularity (measured as the number of retweets) (Mahdikhani 2022). By extracting a different set of content features, the authors show that tweets with higher emotional intensity are more popular compared with tweets containing information on the pandemic (Mahdikhani 2022). Only a few studies focus on Italy, although this country was among the first to be severely hit by the pandemic. De Rosis et al. analyze a large set of Italian tweets posted between February 17, 2020 and March 22, 2020 (De Rosis et al. 2021). The authors show significant changes in the trend of the number of positive and negative tweets that they put in relation to measures undertaken by the government, as well as the narrative offered by the media in the period under consideration.

The rationale for this paper is based on previous literature suggesting that Twitter represents a useful tool to study reactions to epidemic events. This paper makes several contributions to the literature. First, other studies usually use a single method to assess the polarity of a text in a sentiment analysis whilst our approach offers a comparison of different methods (see Sect. 3) and also adds an evaluation of text polarity with machine learning classifiers to compare performances obtained for tweets posted before and after the outbreak (see Sect. 5). Second, while many studies give more emphasis to graphical representation (e.g. word-cloud) or tables (e.g. most used words), in this study we utilize statistical and machine learning tools focused on sentiment scores’ trends rather than on the specific content of tweets. We also analyze individual emotions in order to separate the positive part of a score from the negative one and to better understand how these two components behave. Third, we expand the analysis using economic data from the main Italian stock exchange index to find out if the trend in sentiment towards Italy is related to other aspects (e.g. the economy) of the country. To this regard, we observe an association between the time series of sentiment and stock market performance, with sentiment found to serve as an early detection signal (up to eight days earlier) for potential effects on the stock exchange index values. Fourth, our work considers a period of time starting from the end of 2019 to give a more comprehensive representation of the evolution of sentiment towards Italy, i.e., it is not strictly limited to the COVID-19 outbreak period. In this way, we effectively show the impact that the COVID-19 outbreak had on sentiment toward Italy. To the best of our knowledge, the present study is the first one using social media opinions to consider the effects of the COVID-19 outbreak on both the reputation of a country and its economy.

## Impact of the COVID-19 outbreak towards Italy’s reputation

### Data collection

We use lexicon-based sentiment analysis on tweets to evaluate the temporal evolution of the sentiment towards Italy before and during the COVID-19 outbreak. All tweets posted in the period October 2019–May 2020, in English language and reporting the keyword “Italy" were collected. In total, 4,481,104 tweets were retrieved. After data cleaning, consisting of quality control and removal of tweets for which incomplete content was downloaded, 4,480,788 tweets were retained and used for further analysis.

### Evolution of the sentiment towards Italy before and during the COVID-19 outbreak

**Fig. 1:**
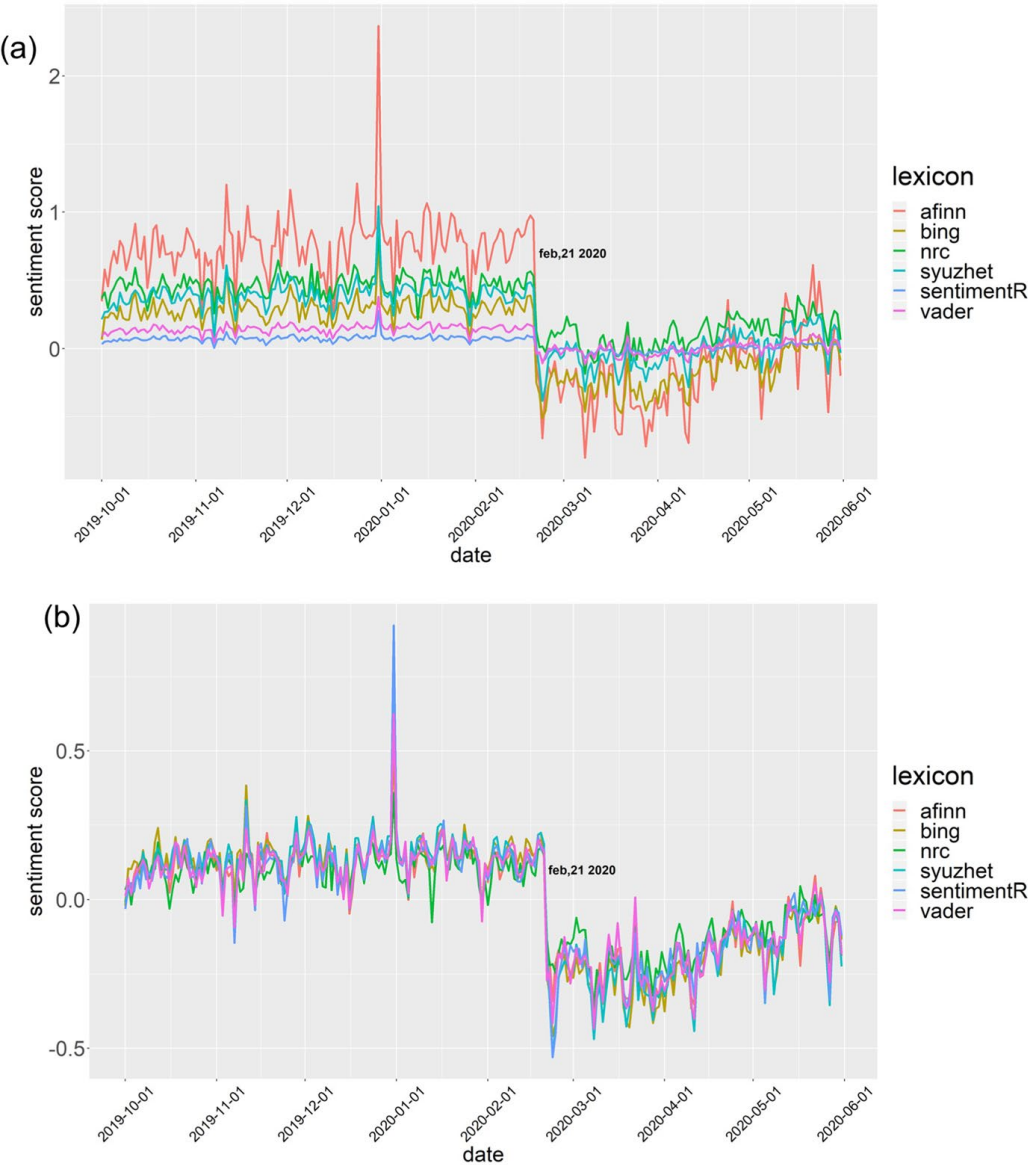
Sentiment score (**a**) and standardized sentiment score (**b**) of collected tweets including the keyword “Italy" from October 2019 to May 2020

Preprocessing of tweets (including removal of punctuation marks, hashtags, mentions, and links as well as conversion in lower case letters) was conducted in R (R Core Team 2021) version 4.0.3. Six different methods were used to evaluate the sentiment of collected tweets: sentimentR (Rinker 2019), vader (Hutto and Gilbert 2014) and four lexicons (nrc, afinn, bing and syuzhet) included in the Syuzhet package (Jockers 2015). For each method, we consider the mean score for tweets collected in a single day. These values were then plotted to observe the temporal evolution of the sentiment toward Italy (Fig. 1a). The mean scores were then standardized to consider the different scales used by each method to report the sentiment of a tweet (Fig. 1b).

As shown in Fig. 1b, a high concordance among sentiment scores assigned by different methods is observed for standardized sentiment scores. The most positive values, corresponding to the highest peak, are observed on New Year’s Eve. Most interestingly, while the trend of sentiment values in the previous months is somewhat stable around neutral or slightly positive values, extremely negative scores are observed from February 21, 2020. On this date, the first Italian case of COVID-19 is reported. From this day, sentiment scores remain negative, although a slow trend towards less negative/neutral values seems to be present in May.

We performed an analysis aimed at finding a structural change in order to verify the existence of a breakpoint in sentiment scores on February 21, 2020. A structural break is a sudden change of values in a time series occurring at one or more specific dates. Therefore, this analysis allows us to gain further insights into the phenomenon object of study by knowing when a significant change occurred in the data. Using the strucchange R package (Zeileis et al. 2002) we conduct the structural change analysis for all six used lexicons.

The strucchange R package estimates breaks in time series regression models, assuming that there are *m* breakpoints where the coefficients shift from one stable regression relationship to a different one and therefore ${m + 1}$ segments in which the regression coefficient are constant. The optimal number of breakpoints is estimated by minimizing both the Bayesian Information Criterion (BIC) and the residual sum of squares (RSS). The function breakpoints estimates multiple breakpoints simultaneously based on the algorithm described in Bai and Perron (2003).

**Fig. 2:**
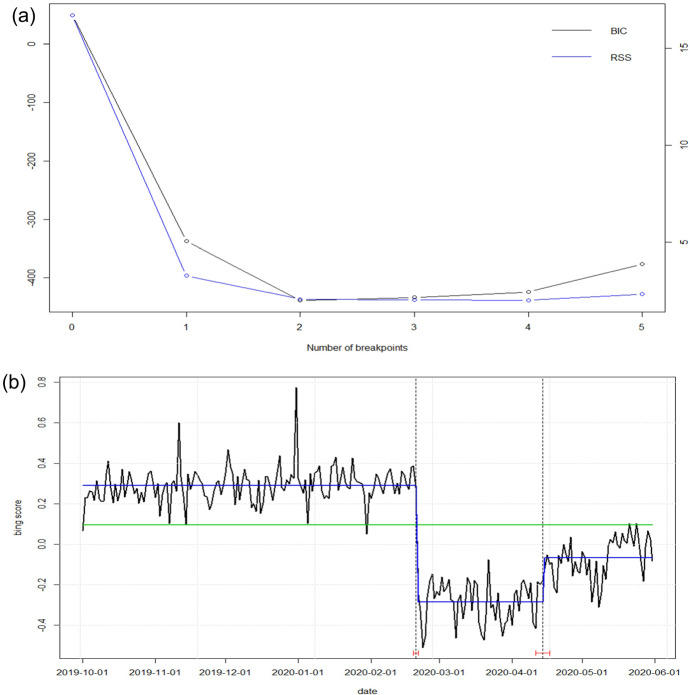
**a** BIC and Residual Sum of Squares using the bing lexicon, **b** Breakpoints in sentiment score using the bing lexicon

The optimal number of breakpoints was two for all lexicons (Table 1 and Fig. 2). For all lexicons, the existence of a breakpoint on February 21, 2020 is observed (Table 1). While we observed variability in the break dates identified for the second breakpoint, for all lexicons this breakpoint corresponded to a trend towards more positive values started in April (Table 1).

**Table 1:** Identified break dates using different lexicons

Lexicon	Break dates	
Afinn	February 21	April 20
Nrc	February 21	April 11
bing	February 21	April 13
syuzhet	February 21	April 12
vader	February 21	April 13
sentimentR	February 21	April 12

**Fig. 3:**
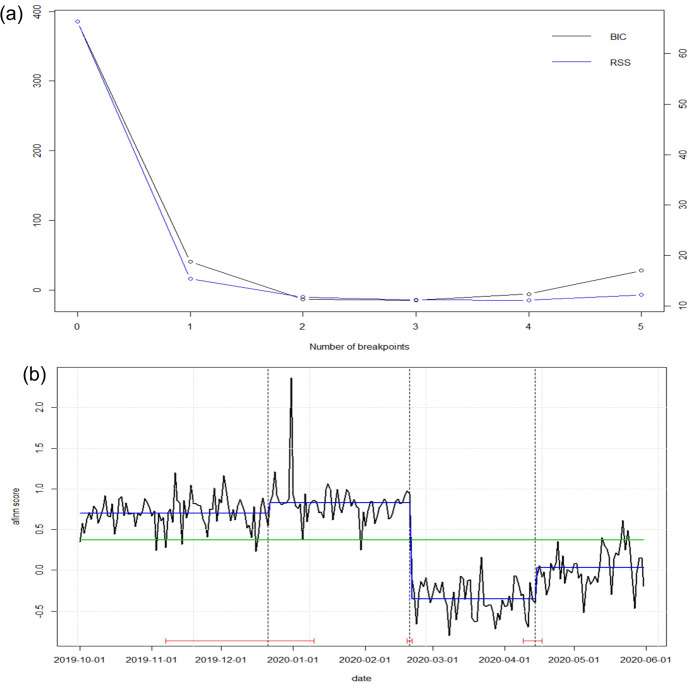
Boxplots showing positive and negative sentiment in Period A (October 1, 2019–20 February 2020) and Period B (21 February–31 May 2020)

**Fig. 4:**
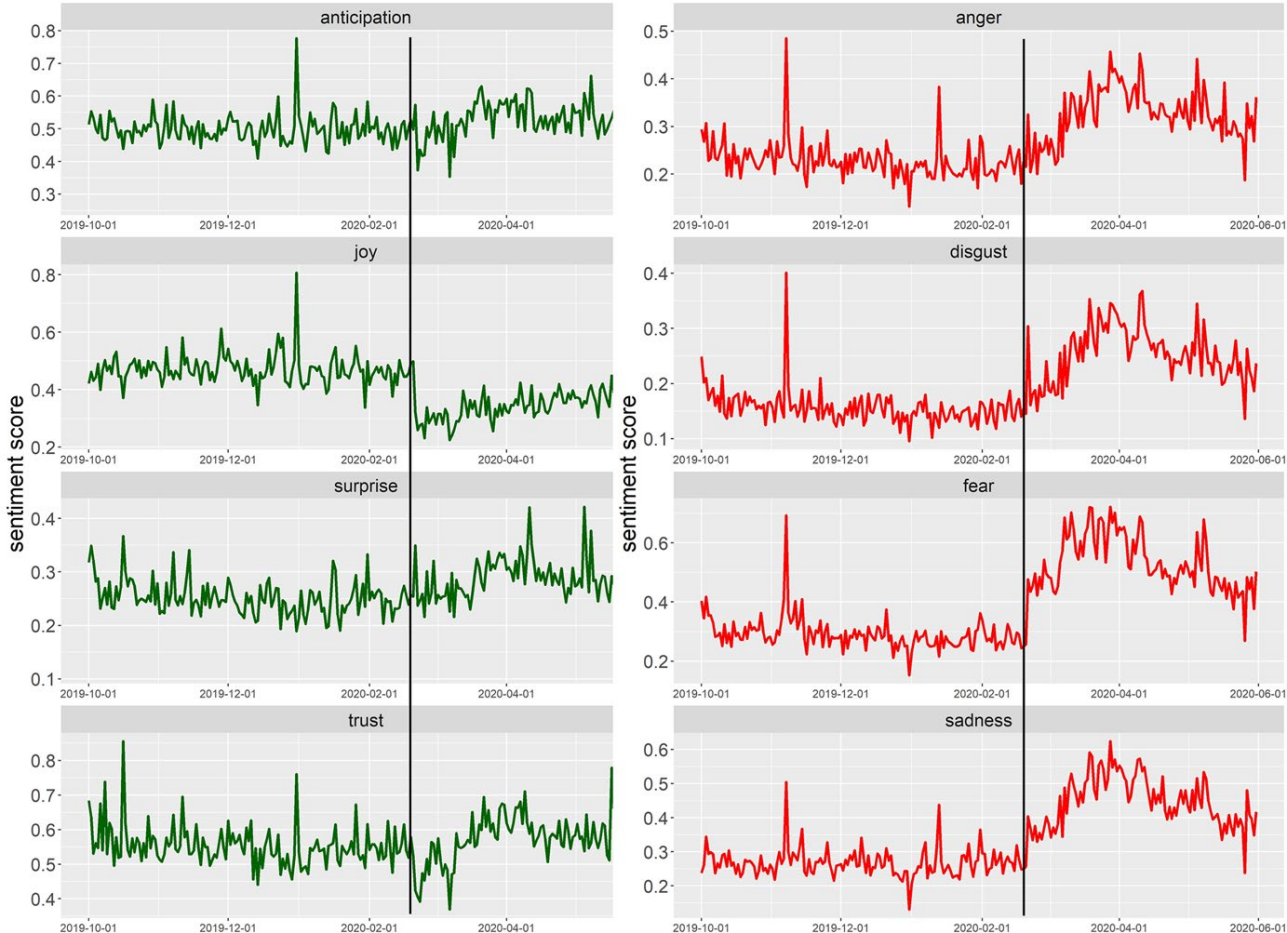
Boxplots showing positive and negative emotions in Period A (October 1, 2019–20 February 2020) and Period B (21 February–31 May 2020)

As we assess that on the date of February 21, 2020, a substantial change of sentiment is observed according to all lexicons, we define two different periods for the subsequent analyses: Period A from October 1, 2019 to February 20, 2020; and Period B from February 21, 2020, to May 31, 2020. Next, we conduct a sub-period analysis of specific positive and negative emotions in these two periods using the nrc lexicon. Besides reporting positive and negative sentiment, this lexicon allows us to evaluate each tweet in terms of eight basic emotions: four negative emotions (anger, disgust, fear, and sadness) and four positive ones (anticipation, joy, surprise, and trust). The scores for positive and negative sentiment in the two Periods A and B are compared in Fig. 3, while scores for the eight specific emotions are shown in Fig. 4. The scores obtained for sentiment as well as for specific emotions show non-normal distribution according to the Shapiro–Wilk test. Moreover, the Mann–Whitney test is used to compare sentiment and emotions between Period A and Period B. As shown in Fig. 3, general positive sentiment is not decreasing significantly ($p = 0.84$) from Period A to B, whilst negative sentiment is significantly increasing ($p <0.001$).

Although a rise of negative emotions in Period B is somewhat expected and in accordance with our hypothesis, positive emotions overall remain stable. This finding is further explored and partly confirmed, through the analysis of specific emotions. As shown in Fig. 4, all negative emotions are increasing from Period A to Period B ($p < 0.001$). As for positive emotions, joy is decreasing significantly ($p <0.001$), whilst anticipation, surprise, and trust are increasing significantly ($p < 0.001$). A decrease in joy can be expected during such a hard time, whilst the other three emotions might increase for different reasons. The rising in anticipation and surprise might be interpreted as follows: even if the COVID-19 outbreak is a negative event, it has the power to generate surprise and to increase the desire to know what will happen in the near future. On the other hand, the increase in trust might depend on the willingness to believe in a speedy recovery as well as on the attitude of people living outside of Italy towards encouraging Italians.

**Fig. 5:**
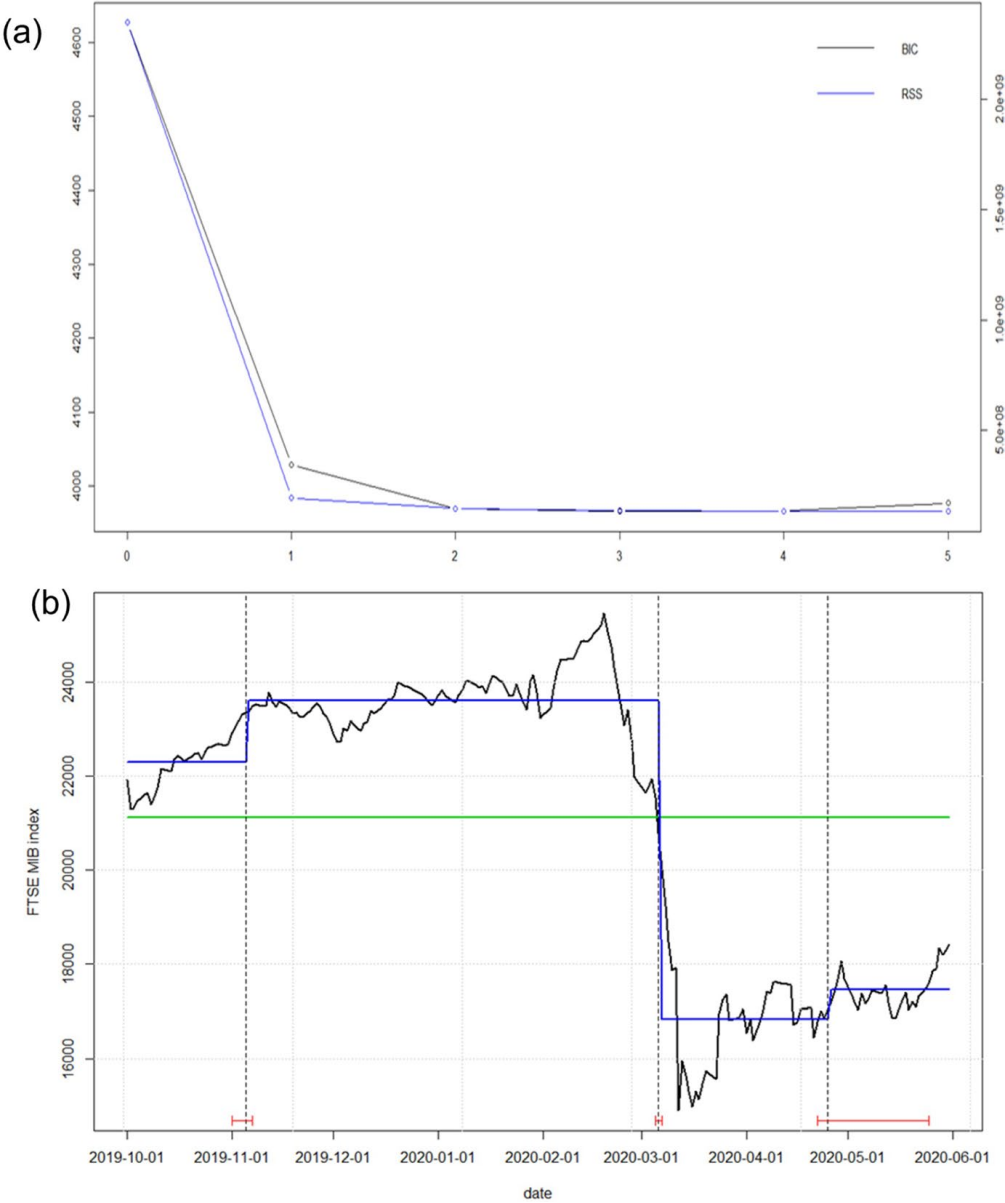
Temporal evolution of the positive (on the left: anticipation, joy, surprise, and trust) and negative (on the right: anger, disgust, fear, and sadness) emotions from October 1 to May 31

Day-to-day detailed temporal evolution for the eight emotions from October 1 to May 31 is shown in Fig. 5. We observe that, from February 21, 2020, all negative emotions (right panel) start to rise, whilst positive emotions (left panel) behave differently: surprise rises, joy first decreases and then remains stable, whilst anticipation and trust first decrease and subsequently show a progressive increase in the last days. Figure 5 gives us the chance to grasp subtle nuances not evident in the general trend but which emerge when detailing the various emotions. The rise in the levels of negative emotions is expected since an event such as the outbreak of an epidemic disease certainly has the power to increase anger, disgust, fear, and sadness both for those who experience the event firsthand and for those who were not directly affected by it at that time. Positive emotions behave differently but consistently with what could be expected of any specific emotion. For example, joy decreases, and it no longer reaches the values of Period A, while trust has a similar huge drop, but it recovers in a very short time (about a month). This is probably due to the fact that at the time people wanted to express their trust that things would get better for Italy. Slogans as *“Andrá tutto bene"* (it will be okay) were very popular in Italy during the spread of the pandemic.

## Analysis of FTSE-MIB index from October 2019 to May 2020

The concept of sentiment in the financial literature has been defined in different ways. Among the most interesting applications, investor sentiment is a research area of behavioral finance aimed at evaluating the sentiment of investors and the way in which it can affect the stock market (Ángeles López-Cabarcos et al. 2020). Investor sentiment has been shown to affect the cross-section of stock returns, with larger effects on securities characterized by subjective and difficult-to-arbitrage valuations (Baker and Wurgler 2006). In the last few years, this research area has attracted high interest, as shown by a recent bibliometric analysis (Paule-Vianez et al. 2020). This can be partly explained by the availability of new instruments to obtain a large body of information to evaluate sentiment, such as microblogging services (Ángeles López-Cabarcos et al. 2020).

**Fig. 6:**
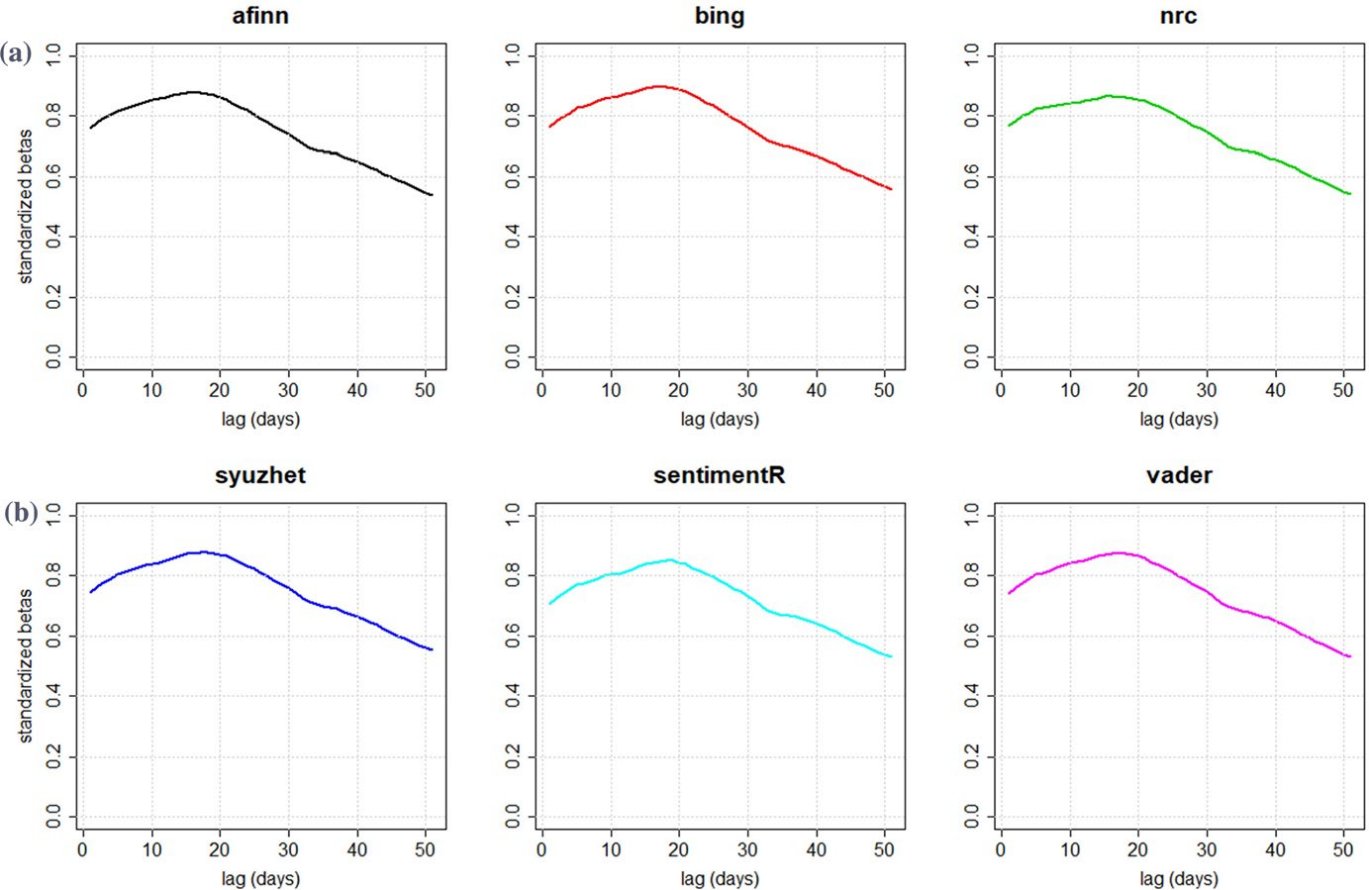
**a** BIC and Residual Sum of Squares using the FTSE-MIB index values; **b** breakpoints in FTSE-MIB index values

To verify whether a trend similar to the one observed for the tweets’ sentiment is also observed for changes in stock exchange value movements, data from the main Italian stock exchange index (FTSE-MIB) are collected. Closing values of FTSE-MIB are observed from October 1, 2019, to May 31, 2020. Values concerning weekends and other festive days are linearly interpolated to be able to compare homogeneously trends of tweets’ sentiments and stock exchange values. The analysis performed on FTSE-MIB evidences the existence of three breakpoints on November 6, 2019, March 7, 2020, and April 26, 2020 (Fig. 6). Specifically, the first breakpoint observed during the COVID-19 outbreak period dates March 7, 2020: it happens 15 days after the change in sentiment scores (February 21, 2020). Interestingly, the day after the observed breakpoint the Lombardy region was set into lockdown.

We also investigate about a possible association between sentiment scores and stock exchange index values. Since we observe a time lag between the main breakpoint in sentiment scores and the corresponding breakpoint in FTSE-MIB values, we hypothesize that this association might be present even when applying a time lag (i.e. past sentiment scores are associated with stock exchange index values in the next days). In order to analyze the relationship between the change of sentiment and FTSE-MIB index values over time, we construct a vector autoregressive (VAR) model using the vars package in R (Pfaff 2008). To evaluate if the association between sentiment scores and FTSE-MIB prices depends on the specific method used to perform sentiment analysis, we estimate the model separately for each method and each lexicon used to compute sentiment scores. First, differences of log-transformed data are taken to make the time series stationary. Stationarity is checked via inspection of the time series plot as well as with the Augmented Dickey–Fuller test implemented in the tseries R package (Trapletti and Hornik 2021). The optimal lag length for the sentiment computed using each lexicon is estimated using the VARselect function of the vars R package, based on the Akaike Information Criterion (AIC) criterion. The stability of the models is checked based on the eigenvalues of the companion coefficient matrix using the vars package. The bidirectional association between sentiment and FTSE-MIB index values is analyzed using the Granger-causality test.

Using the VARselect function, we observe different optimal lags in the relationships between FTSE-MIB index values and sentiment scores computed using the six dictionaries (Table 2).

We then conduct a Granger-causality test on the VAR models estimated using these time lags. The null hypothesis for this test is that lagged values of a variable *X* that evolves over time (i.e. sentiment scores) do not explain the variation in variable *Y* (i.e. FTSE-MIB index values). In other words, variable *X* Granger-causes variable *Y* in case predictions of the values of *Y*, based on its own past values and on the past values of *X*, are better than predictions of the values of *Y* based only on past values of *Y*. We find that FTSE-MIB index values do not Granger-cause sentiment computed using any dictionary. Conversely, sentiment scores computed with afinn and vader are found to Granger-cause FTSE-MIB index values and a trend is observed for NRC (Table 2). While both the drop in sentiment scores as well as in FTSE-MIB values are caused by the pandemic, our results suggest that sentiment scores computed with afinn and vader are useful to predict FTSE-MIB index values with an optimal lag of 8 and 5 days, respectively.

**Table 2:** Optimal time lags and results of the Granger-causality test

Lexicon	Optimal lag (days)	Sentiment G-causes	FTSE-MIB G-causes
		FTSE-MIB	Sentiment
Afinn	8	0.025	0.439
Bing	4	0.135	0.171
NRC	6	0.051	0.463
Syuzhet	8	0.085	0.919
SentimentR	4	0.224	0.640
Vader	5	0.034	0.427

*G-causes* Granger-causes

Overall, these findings enforce the idea that a change in sentiment scores can be considered as an early detection signal (up to eight days earlier) for potential effects on the stock market values.

## Evaluation of tweets’ sentiment using machine learning classifiers

Finally, we try to further investigate whether the difference in the content of tweets posted in the two periods might also affect the performance of two machine learning classifiers widely used in the analysis of Twitter’s data, i.e. NB and SVM, in the prediction of their polarity. Considering that, after the outbreak, the topic was largely discussed on Twitter, we aim to evaluate whether the two classifiers show differences in performance when analyzing texts pertaining to more diverse topics (whole data set and Period A) or a more homogeneous topic (Period B). The main aim of this analysis is to verify whether more homogeneous content (related especially to negative tweets) might lead to a better performance in the classification task in Period B compared with Period A. To this aim, we randomly sample 10,000 tweets (5000 from Period A and 5000 from Period B, respectively) and manually label them into “positive", “neutral" or “negative" according to their specific content. Tweets consisting only of hashtags, URLs, emoticons, or single words are excluded. Of these, 6409 tweets are labeled as positive, neutral, or negative (3227 from Period A and 3182 from Period B) and used for the subsequent analysis. Tweets were manually labeled by a single rater. For a subsample of 20% of the 6409 tweets ($n = 1282$, of which 645 from Period A and 637 from period B) we verified the agreement rate with two independent raters (expert raters from Amazon Turk). We found high concordance between our rating and the one provided by independent raters. Specifically, for 94% of the 645 tweets from Period A and 95% of the 637 tweets from Period B we found an agreement with at least one independent rater. NB and SVM are estimated using the e1071 (Meyer 2019) and caret (Kuhn 2008) R packages. For both NB and SVM the optimal parameters are fine-tuned using cross-validation.

These tweets are first preprocessed (removal of punctuation, numbers and stop words) and used to form a document text matrix using the tm R package (Feinerer and Hornik 2019). For both machine learning classifiers, the analyses are conducted on the whole data set of 6409 positive, neutral, or negative tweets, as well as on the two subsets pertaining to Periods A and B. As the specific content of the negative tweets is more related to COVID-19 in Period B, we expect the two classifiers to perform better for negative tweets posted during this period compared with negative tweets posted during the pre-outbreak period (Period A) or tweets related to the other categories.

For the sake of completeness, in the following, we summarize the main features of NB and SVM.

### Naïve Bayes

Naïve Bayes (NB) classifiers are supervised learning algorithms used for more than two decades for different classification problems, including spam filtering and text classification (Sahami et al. 1998). A NB classifier is based on the Bayes theorem which states that, given two events A and B, we can compute the conditional probability of A given B as the probability of B given A times the probability of A, all divided by the probability of B. Notationally, we have:

1 $$\begin{aligned} P\left( A\big |B\right) =\ \frac{P(B|A)\ P(A)\ }{P(B)} \end{aligned}$$

A NB classifier assumes that features are conditionally independent thus, considering a categorical response variable y with k classes $(k\ge 2)$ the task is to assign each tweet $t_i\left( i=1,\ldots .,n\right)$ to one of the k classes of y. In our case, tweets can be classified either in positive or negative ($k = 2$).

Therefore, any tweet $t_i$ is classified into one, and only one, of the $C_k$ classes.

### Support vector machine (SVM)

SVM is a non-probabilistic binary linear classifier (Suykens and Vandewalle 1999), as it is not based on a specific probability distribution. In a linear setting, SVM detects a line, so-called hyperplane, which best separates data. As in the case of NB, the task is to assign each tweet $t_i\left( i=1,\ldots .,n\right)$ to one of the two classes (positive or negative) of the categorical response y. The hyperplane that best splits the data is that positioned as far as possible from the nearest points of the two classes. The distance between a point and the hyperplane is called the margin. The larger these margins (hard margins), the smaller the probability of misclassification error. To train a linear SVM, we consider data $(\vec {x}_1, y_1), \ldots , (\vec {x}_n, y_n)$, where $\vec {x}_i$ are vectors of feature values of observation $i\ \left( i=1,\ldots .,n\right)$, and $y_i$ are labels (+1 or -1, depending on which class an observation belongs to). The goal is to find a hyperplane

2 $$\begin{aligned} \vec {w} \cdot \vec {x}_i + b = 0 \end{aligned}$$

that best separates the two classes, with $\vec {w}$ being the normal vector to the hyperplane. One of the main problems that may arise when looking for the best hyperplane is that even a single new observation might cause a great shift in the hyperplane, reducing the margin by a great amount. In such cases, it is preferable to have a classifier able to tolerate a sub-optimal separation of the two classes but apt to deliver a higher overall classification performance (soft margins). In practice, some points are allowed to be on the wrong side of the margin, or even of the hyperplane, but only up to a given distance less or equal to a constant value.

### Comparison between the performance of Naïve Bayes and support vector machine

For both models, the k-fold cross-validated ($k = 5$) performance in the prediction of sentiment is reported in Table 3. With respect to the whole test set (Period A + Period B), NB reaches a good accuracy for negative and positive tweets (0.77 and 0.78, respectively) and a lower accuracy for neutral tweets (0.66). A similar difference is observed for sensitivity and positive predictive value (PPV), for which good performance is only observed for positive and negative tweets. Conversely, specificity is high for all examined classes. Restricting the analysis to Period A, the classifier shows a generally worse performance, especially in the classification of positive and negative tweets. We can speculate that this worse performance might be related to a more heterogeneous content of tweets posted in this period. However, it cannot be excluded that this performance might simply be related to the limited ability of the classifier to correctly identify the polarity of tweets due to limited sample size of the training data set. Indeed, the reported results have to be carefully interpreted in light of the small number of labeled tweets as well as the variability observed across metrics. An opposite result is observed when restricting to Period B. For negative tweets, all metrics show a small improvement compared to either Period A or the whole data set (Table 3), while more variable results can be observed for neutral and positive tweets. Again, this result has to be interpreted in light of the limited number of tweets used to train the classifier. While these results have to be interpreted in light of this limitation, we found that NB shows an improvement in the classification accuracy when analyzing a data set in which a specific topic, i.e. the COVID-19 pandemic, characterizes the content of a large part of negative tweets (Period B) compared to periods in which more diverse topics are discussed by users (whole data set and Period A).

**Table 3:** Fivefold cross-validated performance of Naïve Bayes and support vector machine in the classification of tweets

	NB			SVM		
	Positive	Neutral	Negative	Positive	Neutral	Negative
*Both periods*						
Sensitivity	0.72 (± 0.03)	0.53 (± 0.03)	0.69 (± 0.02)	0.67 (± 0.02)	0.66 (± 0.02)	0.67 (± 0.03)
Specificity	0.83 (± 0.02)	0.79 (± 0.02)	0.86 (± 0.01)	0.88 (± 0.01)	0.74 (± 0.01)	0.89 (± 0.01)
PPV	0.69 (± 0.02)	0.52 (± 0.02)	0.73 (± 0.02)	0.73 (± 0.01)	0.53 (± 0.01)	0.78 (± 0.02)
NPV	0.86 (± 0.01)	0.79 (± 0.01)	0.83 (± 0.01)	0.84 (± 0.00)	0.83 (± 0.01)	0.83 (± 0.01)
F1-score	0.70 (± 0.01)	0.53 (± 0.02)	0.71 (± 0.02)	0.70 (± 0.01)	0.59 (± 0.01)	0.72 (± 0.03)
Accuracy	0.78 (± 0.01)	0.66 (± 0.01)	0.77 (± 0.01)	0.77 (± 0.01)	0.70 (± 0.01)	0.78 (± 0.02)
*Period A*						
Sensitivity	0.63 (± 0.03)	0.58 (± 0.05)	0.63 (± 0.03)	0.58 (± 0.05)	0.74 (± 0.04)	0.56 (± 0.03)
Specificity	0.84 (± 0.03)	0.76 (± 0.02)	0.82 (± 0.03)	0.90 (± 0.02)	0.65 (± 0.03)	0.89 (± 0.03)
PPV	0.68 (± 0.06)	0.52 (± 0.03)	0.65 (± 0.03)	0.76 (± 0.04)	0.49 (± 0.04)	0.73 (± 0.05)
NPV	0.81 (± 0.01)	0.81 (± 0.03)	0.81 (± 0.01)	0.80 (± 0.02)	0.85 (± 0.02)	0.79 (± 0.02)
F1-score	0.65 (± 0.03)	0.55 (± 0.03)	0.63 (± 0.00)	0.66 (± 0.03)	0.59 (± 0.03)	0.64 (± 0.03)
Accuracy	0.74 (± 0.02)	0.67 (± 0.03)	0.72 (± 0.00)	0.74 (± 0.02)	0.70 (± 0.02)	0.73 (± 0.02)
*Period B*						
Sensitivity	0.73 (± 0.02)	0.46 (± 0.03)	0.73 (± 0.04)	0.69 (± 0.03)	0.59 (± 0.05)	0.69 (± 0.04)
Specificity	0.81 (± 0.02)	0.81 (± 0.01)	0.86 (± 0.01)	0.86 (± 0.02)	0.74 (± 0.03)	0.90 (± 0.02)
PPV	0.65 (± 0.02)	0.51 (± 0.02)	0.76 (± 0.02)	0.70 (± 0.02)	0.50 (± 0.03)	0.81 (± 0.04)
NPV	0.86 (± 0.02)	0.78 (± 0.02)	0.84 (± 0.02)	0.85 (± 0.01)	0.81 (± 0.02)	0.83 (± 0.02)
F1-score	0.69 (± 0.02)	0.48 (± 0.02)	0.74 (± 0.03)	0.69 (± 0.02)	0.54 (± 0.04)	0.74 (± 0.03)
Accuracy	0.77 (± 0.02)	0.64 (± 0.01)	0.79 (± 0.03)	0.77 (± 0.02)	0.66 (± 0.03)	0.79 (± 0.02)

We report mean values (standard deviation) for 5-fold cross validation

*NPV* negative predictive value, *PPV* positive predictive value. More information in the Appendix

Next, SVM with a radial kernel is estimated in the same way as NB. Again, when analyzing the whole data set (Period A + Period B) the classifier shows a good accuracy, specificity and PPV for positive and negative tweets, but a worse performance for neutral tweets (Table 3). When analyzing Period A, consistent with the results obtained with NB, SVM shows lower accuracy in the classification of positive and negative tweets. Conversely, higher sensitivity and negative predictive value (NPV) are obtained for neutral tweets. When analyzing Period B, as observed with NB, the classification of negative tweets improves based on all metrics, while the classification of neutral and positive tweets generally shows a lower performance compared with the whole data set. Again, the performance of this classifier might be affected by the small number of tweets that were labeled in our study, which might have especially affected SVM as non-parametric machine learning methods usually require large data sets to obtain accurate predictions.

Results obtained for the whole data set (Period A + B, Table 3) evidence that the two classifiers show similar performance based on all metrics except sensitivity, for which is better but still not ideal results for the neutral class are obtained with SVM.

For the tweets collected during Period A, SVM shows a higher sensitivity in the classification of neutral compared with positive and negative tweets, while the opposite is observed with NB. However, overall the observed differences are small and none of the classifiers shows an adequate performance in this task. Accuracy and NPV are similar for all classes and for both models. When analyzing the tweets collected during Period B it can be observed that, for both models, the classification of negative tweets shows better performances compared with other classes as well as with negative tweets related to Period A or the whole data set. However, for some of the metrics these differences were small in nature, while more evident differences could be observed for PPV and F1-score.

Overall, these results might support our initial assumption that classifiers’ performance is improving for negative tweets following the beginning of the outbreak, as the content of tweets is more specifically focused on the pandemic. This was especially true for negative tweets, as we can speculate their content to be particularly homogeneous and mostly related to the pandemic.

Based on the small number of labeled tweets, our analysis might not have enough power to show high performance in the task of classification of single tweets and this was especially true for the neutral class, for which we observed a generally worse performance. This is probably due to the fact that neutral tweets can contain mixed feelings about the object of study or ambiguous statements hard to interpret, which can therefore lead to a higher number of classification errors. Although the two classifiers show small differences with regard to the classification of neutral tweets, for which SVM showed a slightly better performance in terms of sensitivity, we did not observe differences with a magnitude that might lead to the choice of one of the two classifiers over the other.

## Concluding remarks

In this paper, we analyze the sentiment towards Italy before and after the COVID-19 outbreak using lexicon-based and machine-learning classifiers applied to real data collected from Twitter. We observe a substantial rise in negative emotions towards Italy in correspondence to the first Italian case of COVID-19 followed by a change towards more neutral or slightly positive values starting two months later. Besides being useful to interpret the general sentiment towards a country as a proxy of the perceived country’s reputation, we find that sentiment scores can be also used to early detect changes in stock exchange values. Future research is addressed to assess the sentiment towards different countries, to verify whether similar findings might be observed also in cases in which the outbreak developed with different rates of escalation and/or in cases when severity is managed by the governments with a different alternative or concomitant measures such as social distancing, lockdown or travel restrictions. The results of this research must be interpreted in the context of its limitations. First, data were collected from a single social media (i.e., Twitter). It is possible that the results could vary in the case many social media are considered. However, as shown in the literature review, Twitter is widely used to evaluate reactions to important events due to its diffusion and ease of use. In addition, since we only analyzed tweets in English, our results might be influenced by an Anglocentric view of Italy.

As already mentioned, future work should also focus on other countries, as our findings could vary due to the different country’s reputations or other factors (e.g. cultural or socioeconomic factors). Despite these limitations, we believe that this analysis is helpful to understand how the sentiment towards Italy, one of the first countries severely affected by COVID-19, evolved over time. Furthermore, it can also help to shed light on the relationship between country’s reputation and the possible economic repercussions of an event of this magnitude.

## Appendix

Below we clarify how the metrics used in Sect. 5.3, specifically in Table 3, were computed.

See Table 4.

**Table 4:** Definition of the metrics used to assess the performance of NB and SVM in the classification of tweets

Metric name	Definition
Sensitivity	$\frac{TP}{TP+FN}$
Specificity	$\frac{TN}{TN+FP}$
PPV	$\frac{TP}{TP+FP}$
NPV	$\frac{TN}{TN+FN}$
F1-score	$2\cdot \frac{PPV \cdot Sensitivity}{PPV + Sensitivity}$
Accuracy	$\frac{TP+TN}{TP+TN+FP+FN}$

$TP =$ True Positive: number of correctly classified true cases ($y_i=1$)

$TN =$ True Negative: number of correctly classified negative cases ($y_i=0$)

$FP =$ False Positive: number of incorrectly classified positive cases

$FN =$ False Negative: number of incorrectly classified negative cases

$NPV =$ Negative Predictive Value

$PPV =$ Positive Predictive Value
