# Correction to: Using sentiment analysis to evaluate the impact of the COVID-19 outbreak on Italy’s country reputation and stock market performance

**DOI:** 10.1007/s10260-023-00701-5

**Published:** 2023-05-16

**Authors:** Gianpaolo Zammarchi, Francesco Mola, Claudio Conversano

**Affiliations:** Department of Economics and Business Science, University of Cagliari, Cagliari, Italy

**Correction to: Statistical Methods & Applications** 10.1007/s10260-023-00690-5

In the published version of the paper, two figures have been uploaded from a previous version. In view of that, the captions of Figs. 3, 4, 5 and 6 are correct, but the reported figures do not correspond to the captions.

We report below Figs. 3, 4, 5 and 6 as they would have appeared in the correct version of the paper.

**Fig. 3:**
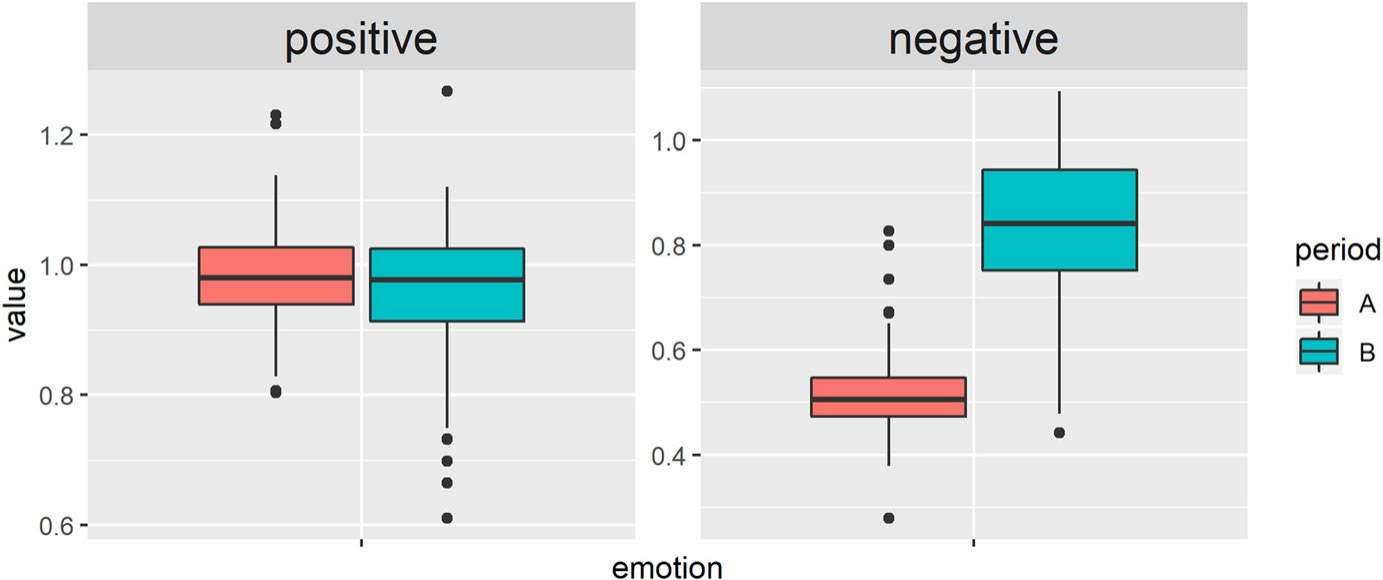
Boxplots showing positive and negative sentiment in Period A (October 1, 2019–20 February 2020) and Period B (21 February–31 May 2020)

**Fig. 4:**
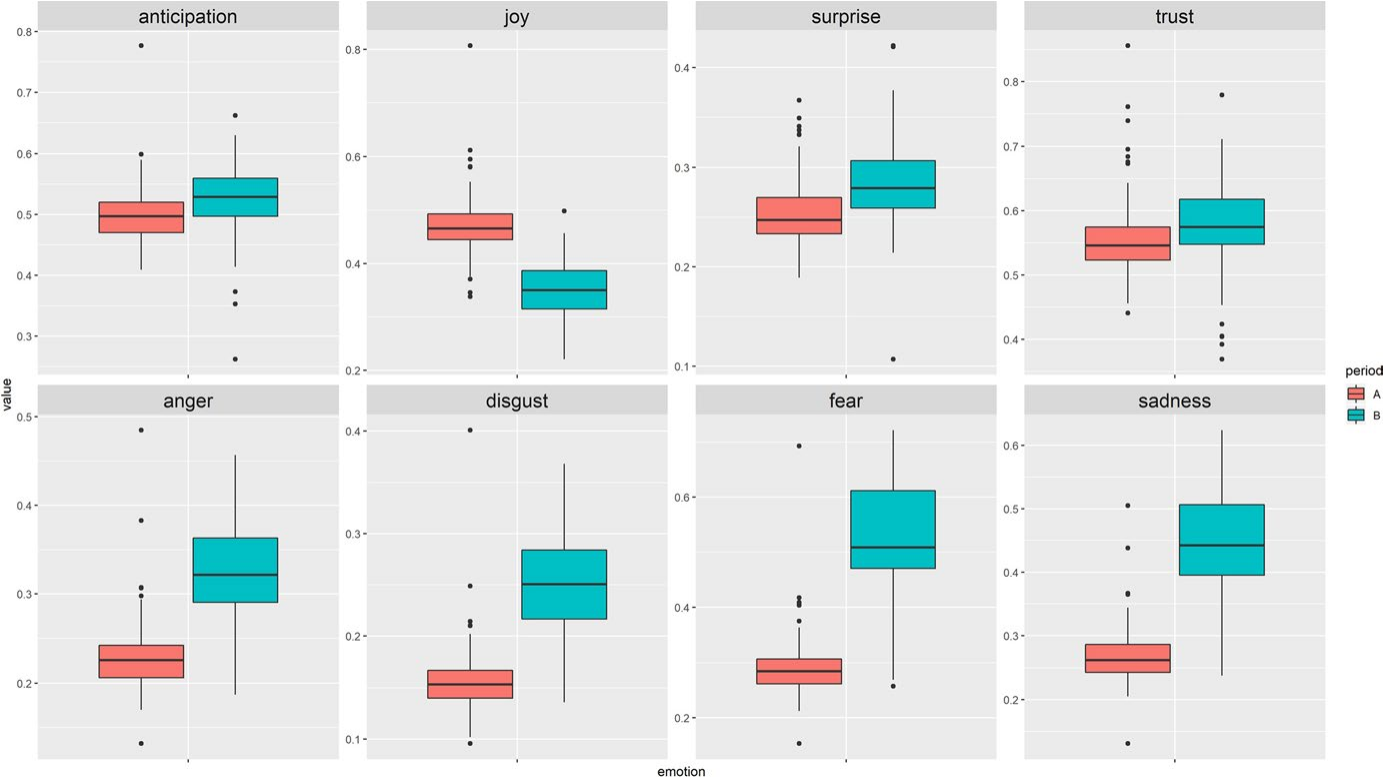
Boxplots showing positive and negative emotions in Period A (October 1, 2019–20 February 2020) and Period B (21 February–31 May 2020)

**Fig. 5:**
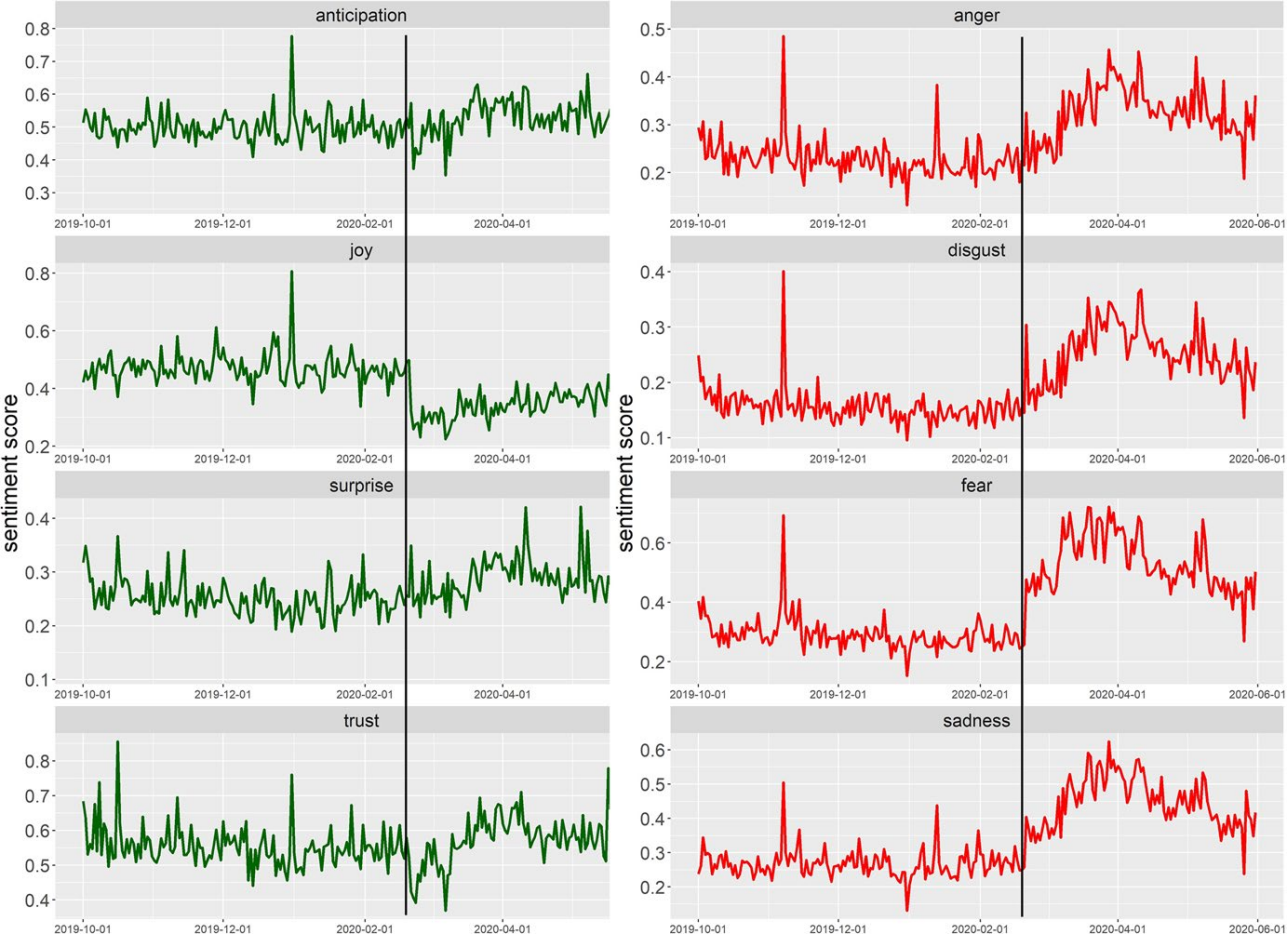
Temporal evolution of the positive (on the left: anticipation, joy, surprise, and trust) and negative (on the right: anger, disgust, fear, and sadness) emotions from October 1 to May 31

**Fig. 6:**
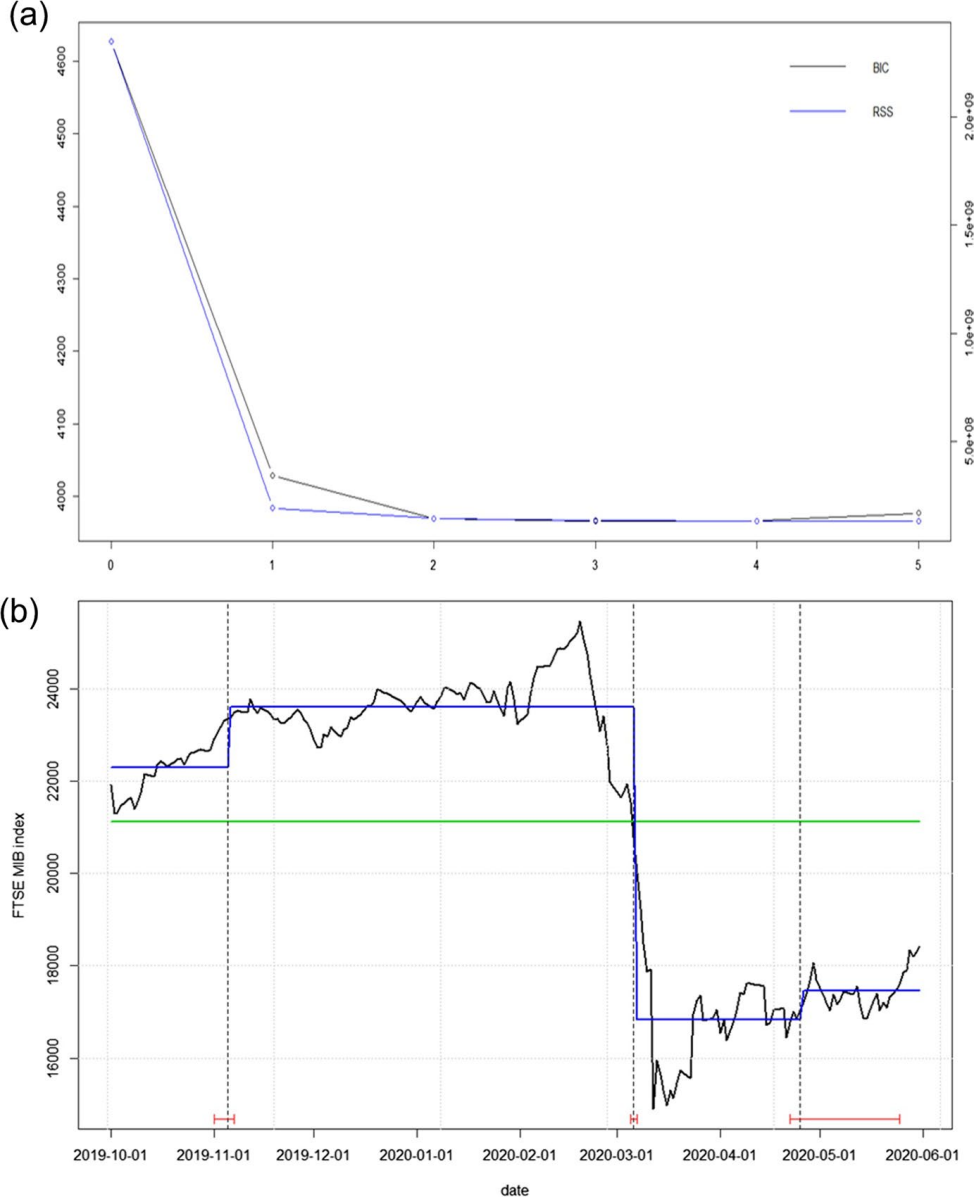
**a** BIC and Residual Sum of Squares using the FTSE-MIB index values; **b** Breakpoints in FTSE-MIB index values

